# Suubi + Adherence4Youth: a study protocol to optimize the Suubi Intervention for Adherence to HIV treatment for youth living with HIV in Uganda

**DOI:** 10.1186/s12889-023-15564-4

**Published:** 2023-04-20

**Authors:** Fred M. Ssewamala, John A. Sauceda, Rachel Brathwaite, Torsten B. Neilands, Proscovia Nabunya, Derek Brown, Ozge Sensoy Bahar, Flavia Namuwonge, Noeline Nakasujja, Allan Mugarura, Abel Mwebembezi, Portia Nartey, Barbara Mukasa, Marya Gwadz

**Affiliations:** 1 International Center for Child Health and Development, Brown School, Washington University in St. Louis, Campus Box 1196, One Brookings Drive, St. Louis, MO 63130 USA; 2 Center for AIDS Prevention Studies, Department of Medicine, University of California, San Francisco, 550 16th Street, San Francisco, CA 94158 USA; 3 Brown School, Washington University in St. Louis, Campus Box 1196, One Brookings Drive, St. Louis, MO 63130 USA; 4 Department of Psychiatry, College of Health Sciences, Makerere University, Kampala, Uganda; 5 International Center for Child Health and Development, Masaka, Uganda; 6 Reach the Youth Uganda, Kampala, Uganda; 7 Mildmay Uganda, Kampala, Uganda; 8 Intervention Innovations Team Lab (IIT-Lab), New York University Silver School of Social Work, New York, NY USA; 9 Center for Drug Use and HIV Research, School of Global Public Health, New York University, New York, NY USA

**Keywords:** Optimization, Suubi + adherence, Youth, Economic empowerment, Viral suppression, Adherence, Intervention components, Multiphase Optimization Strategy, Adolescents living with HIV

## Abstract

**Background:**

Suubi is an evidenced based multi-component intervention that targets psychosocial and economic hardships to improve ART adherence, viral suppression, mental health, family financial stability, and family cohesion for adolescents living with HIV (ALHIV) in Uganda. Suubi was originally tested as a combined package of four components: 1) Financial Literacy Training; 2) incentivized matched Youth Savings Accounts with income-generating activities; 3) a manualized and visual-based intervention for ART adherence and stigma reduction; and 4) engagement with HIV treatment-experienced role models. However, it is unknown if each component in Suubi had a positive effect, how the components interacted, or if fewer components could have produced equivalent effects. Hence, the overall goal of this new study is to identify the most impactful and sustainable economic and psychosocial components across 48 health clinics in Uganda.

**Methods:**

A total of 576 ALHIV (aged 11–17 years at enrollment) will be recruited from 48 clinics and each clinic will be randomized to one of 16 study conditions. Each condition represents every possible combination of the 4 components noted above. Assessments will be conducted at baseline, 12, 24, 36 and 48- months post-intervention initiation. Using the multi-phase optimization strategy (MOST), we will identify the optimal combination of components and associated costs for viral suppression, as well as test key mediators and moderators of the component-viral suppression relationship.

**Discussion:**

The study is a shift in the paradigm of research to use new thinking to build/un-pack highly efficacious interventions that lead to new scientific knowledge in terms of understanding what drives an intervention’s success and how to iterate on them in ways that are more efficient, affordable and scalable. The study advances intervention science for HIV care outcomes globally.

**Trial Registration:**

This project was registered at clinicaltrials.gov (NCT05600621) on October, 31, 2022. https://clinicaltrials.gov/ct2/show/NCT05600621

**Supplementary Information:**

The online version contains supplementary material available at 10.1186/s12889-023-15564-4.

Contributions to the literature

- Most HIV prevention and treatment interventions in Sub-Saharan Africa are “transported” from the Global North, [1–7] whereas our intervention was developed and tested within the Global South’s existing institutions and infrastructure.
- We are addressing structural economic factors that constitute a major gap in HIV intervention science, especially as failure to explicitly address economic hardships will limit the degree to which we can meaningfully improve HIV care outcomes in a sustainable way.
- Our trial design will allow us to identify the key active ingredients and associated costs for a multi-component psychosocial and economic empowerment intervention tested in 48 health clinics in Uganda for adolescents living with HIV with the goal of achieving and sustaining viral suppression.

## Background

A majority of the approximately 3.3 million children and adolescents below 15 years of age who are living with HIV globally are approaching young adulthood [8]. Unfortunately, many of these adolescents living with HIV (ALHIV) live in poverty in sub-Saharan Africa (SSA). A major driver of poor health outcomes and non-adherence to HIV treatment (antiretroviral therapy; ART) are persisting economic and psychosocial hardships in SSA [9–16]. In Uganda, one of the SSA countries hardest hit with HIV with unprecedented numbers of ALHIV (over 170,000) [17, 18]. Yet even with the roll-out of free ART in Uganda which began in 2004 [18], a large and growing number of ALHIV have difficulty managing HIV as a chronic, highly stigmatized, and transmittable illness [19–21]. In Uganda, the prevalence of viral load suppression, a marker of effective treatment, is distinctly lower among HIV-infected young adults aged 15–24 years (57.8% among young women and 43.5% among young men), compared to 93.5% among older women aged 45–54 years and 91% among older men aged 55 to 64 years [22]. Hence, addressing ART non-adherence during the transition to young adulthood is critical [21, 23, 24] for: 1) preventing consequences of viremia and inflammation associated with non-adherence, and 2) reducing risk of becoming resistant to first-line treatments with second-line treatment options being out of reach due to cost and limited availability in SSA.

Adolescence is a challenging transition period [10] critical for identity formation [10, 15] and transition into young adulthood [11] – a period defined in terms of five major role transitions: completing school, leaving home, entering the workforce, forming a romantic partnership, and transitioning to parenthood [25, 26]. During the transition into adult roles and responsibilities [27–29], adolescents have fewer social controls and may establish patterns of positive and risky health behaviors that carry through to adulthood [30–32]. Some of the freedoms young people acquire during this transition encourage exploration and experimentation leading to increased risk-taking behaviors, lower perceptions of social support, and development of mental health problems [11, 33]. Specifically, for ALHIV, this development stage is associated with the lowest ART adherence [34–43] and high risk behaviors [13, 44, 45]. Thus, timely intervention during adolescence can alter negative pathways and optimize health and successful transition to adulthood [27, 46, 47].

Financial instability is another major factor associated with ART non-adherence in multiple ways [36, 43, 48]. For example, when patients initiate ART, increased appetite is one side-effect that requires greater caloric consumption. An increased appetite can have serious implications for impoverished families as studies show fear of not having sufficient food is a barrier to ART adherence [38, 39, 43]. Further, the costs of competing needs and transportation to health clinics leads to missed HIV care visits and refilling ART prescriptions [38, 40]. Importantly, while ART is free in Uganda, financial instability, defined as a lack of assets, monetary income, and material resources, compounds other hardships ALHIV have to deal with. We know that psychosocial interventions alone – the main focus in standard of care—do not eliminate economic hardships that drive poor HIV care and treatment outcomes, to address this the Suubi intervention was designed.

The Suubi intervention is an evidence-based and theory-informed intervention that targets psychosocial and economic hardships and has demonstrated robust effects on viral suppression, ART adherence [49–51], mental health, psychosocial outcomes [52–55], family financial stability [56, 57], and family cohesion [58, 59]. The Suubi intervention was developed, and tested over 15 + years in numerous SSA studies including SEED (2004) [2, 60], Suubi (2005–2008) [61–63], and Suubi-Maka (20,082,012) [53, 64–69], which together informed *Suubi* + *Adherence* (2012–2018) [70]. Given that most HIV interventions for SSA countries have been “transported” from the global north [1, 2, 5, 7, 71], the Suubi Intervention was proven to be culturally relevant for improving ART treatment adherence, reducing HIV-related risk-taking behaviors, and improving mental health outcomes among ALHIV in Uganda. However, the Suubi intervention [1, 72] was tested as a package of four components: 1) Financial Literacy Training (FLT); 2) incentivized matched Youth Savings Accounts (YSA) with income-generating activities (IGAs); 3) a manualized and visual-based intervention for ART adherence and stigma reduction (Suubi Cartoon); and 4) engagement with HIV treatment-experienced role models [70]. Details of Suubi intervention components are described in the methods below. However, it is unknown if each component in Suubi had a positive effect, how the components interacted, or if fewer components could have produced equivalent effects [73–75]. Given our successes and infrastructure, we are well-positioned to unpack and optimize Suubi across Uganda.

Hence, guided by the multi-phase optimization strategy (MOST) framework [73], the overall goal of the Suubi+Adherence4Youth study is to identify the most impactful and sustainable economic and psychosocial components to support sustained viral suppression among ALHIV through testing four Suubi intervention components in an optimization trial. An optimized intervention that is built within existing real-world constraints in SSA for a high-priority group is an innovative and promising way to advance intervention science for HIV care outcomes globally. The proposed optimization of the Suubi intervention is a one-of-a-kind chance to take 15+ years of research developed in SSA and tailor it specifically to improve viral suppression among ALHIV, an important public health need [49–51, 53, 55–58, 61, 63, 76–82].

The primary aims are to: 1) conduct a factorial experiment (optimization trial) to test the main effects of each of the four Suubi intervention components and combinations of components (interactions) on viral suppression (primary outcome); 2) Test mediators and explore moderators that explain and modify the relationship between each of the four Suubi intervention components and viral suppression; and 3) compare the cost and cost-effectiveness of each of the four Suubi intervention components and every combination of components.

## Methods

We referred to the SPIRIT checklist to guide reporting of the study protocol for this clinical trial. (Refer to Supplemental file, SPIRIT Checklist).

### Study setting

The study will be based in Masaka, Rakai, Kyotera, Lwengo, Kalungu and Bukomansimbi, six poor Districts in Southern Uganda hardest hit by HIV and AIDS (prevalence 9.8% vs. 7.3% national average) [83]. The study involves the active collaboration of the Diocese of Masaka, Ugandan Ministry of Health and the health clinics and community-level organizations. Each of the 48 clinics being randomized to one of 16 experimental conditions are located in these districts.

### Partnering sites

The study received support from government extension workers, health workers and local leaders across the study region where we have worked for 15 + years (since 2004). Local AIDS support organizations that the study engaged with include: TASO (one of the oldest AIDS support organizations in Uganda); Villa Maria Hospital, a referral hospital under the Diocese of Masaka who provide community outreach projects for people affected by HIV/AIDS; Rakai Health Sciences Program (RHSP), a research institution but also a site for HIV testing and care; Kituvu Mobile Clinic; Kalisizo Hospital; and Masaka Hospital. Participant recruitment has gone smoothly due to the trust built between community members and our team and collaborators. WUSTL’s ICHAD has established furnished offices with project field staff in place. The study will use these established institutional mechanisms, social support and infrastructure. Reach the Youth-Uganda (RTY-Uganda) also has offices in the study region. Inclusion of RTY-Uganda in the implementation strengthens local implementation capacity for scale-up.

### Framework

The Multiphase Optimization (MOST) is an engineering-inspired framework for building optimized interventions [73]. MOST is not a specific experimental design but a framework to develop, optimize, and evaluate a multi-component intervention. Given our prior intervention research, we are set up to perform an *optimization trial* using a factorial experiment to identify the most cost-effective Suubi intervention components for sustained viral suppression considering three real-world constraints. *Components* are the separate ingredients that go into a packaged intervention. *Optimizatio*n, which is a buzz word in intervention science, must be clearly defined in MOST as it takes inspiration from engineering where performance is evaluated against real-world constraints. *Constraints* are barriers that affect interventions in the real-world. Key constraints in MOST are: 1) economic factors (e.g., time, money, labor), 2) efficiency factors (e.g., how well are resources and staff and participant time and effort allocated) and 3) scalability factors (i.e., degree to which implementation mimic real-world settings). The *optimization objective*, is defined as [73, 74] the most cost-effective combination of *Suubi* components we can achieve within the three real-world constraints defined above. Our optimization objective was chosen to promote the scale up of *Suubi* 2.0 (Suubi + Adherence4Youth) across health systems in Uganda [84].

### Trial design & conceptual model

**Fig. 1:**
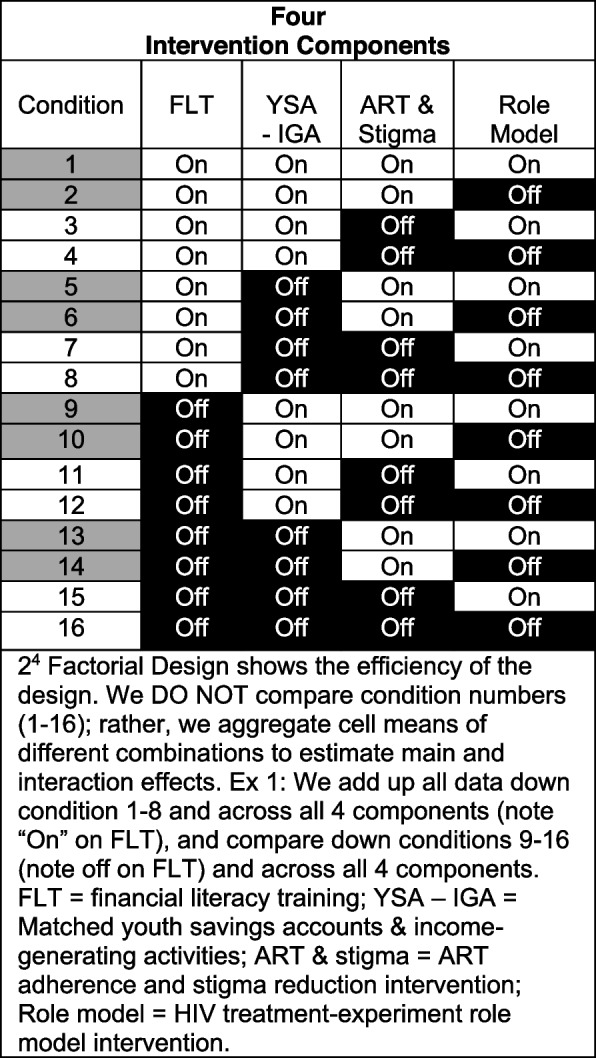
2^4^ Factorial Design

**Fig. 2:**
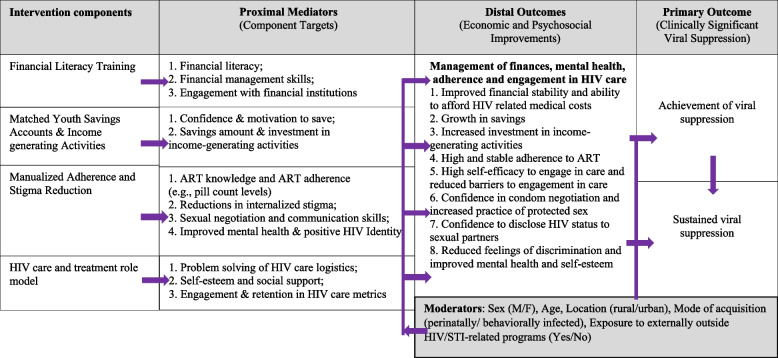
Conceptual Model for Suubi + Adherence4Youth. The model depicts the hypothesized pathway from each component to the intended outcome. It is not a logic nor path model describing every relationship, but instead shows theoretically-informed mediators and how they affect the outcome.

The trial design is a 2^4^ factorial experiment resulting in 16 unique conditions representing all possible combination of 4 components (See Fig. 1). This design will not result in a 16-arm comparison. Rather, the 16 conditions allow us to understand how all 4 components perform. This shows the unique contribution of each intervention component, collapsing over every other component. We can also estimate potential additive, synergistic (multiplicative), or antagonist (multiplicative) effects between components to understand how combinations of components and multiplicative effects of components affect the primary outcome. Figure 2 shows the Conceptual Model which is akin to an engineering drawing in MOST. It specifies the hypothesized *theoretical and empirical* causal processes for each component [73, 85] and gives an “under the hood” look at how causal processes will be falsified or confirmed [73]. The conceptual model guides our secondary analyses around the mediators and moderators of the component-to-viral suppression pathway.

### Randomization

**Fig. 3:**
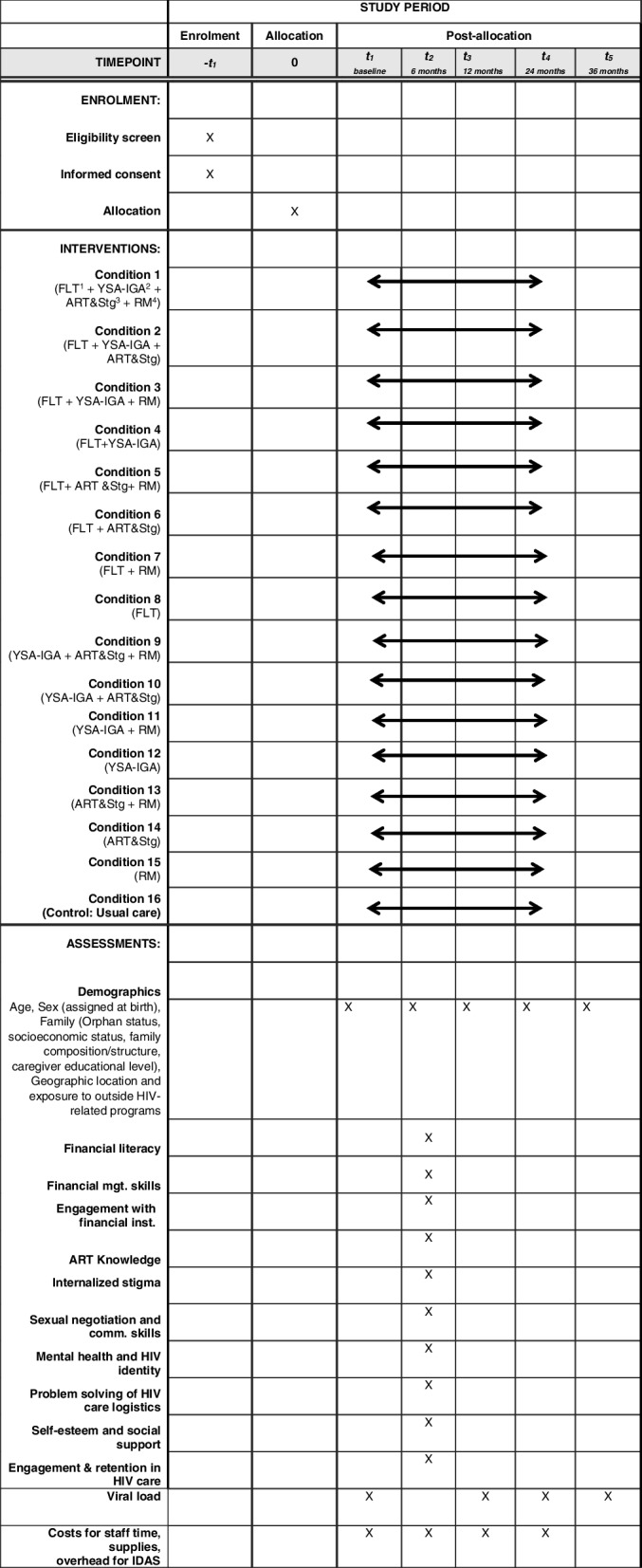
SPIRIT flow diagram: flowchart of the study schedule of enrolment, interventions, and assessments ^1^FLT: Financial Literacy Training workshops. ^2^YSA-IGA: Incentivized matched Youth Savings Accounts (YSA) with income-generating activities (IGAs). ^3^A manualized visual-based intervention for ART adherence and stigma reduction using multiple family group approach (Suubi Cartoon). ^4^RM: Role Models (also known as treatment buddies)

Aim 1 is a 2^4^ factorial experiment resulting in 16 unique conditions representing all possible combinations of the 4 components. Randomization will be at the level of health clinics (*N* = 48 and geographically distant from each other) to prevent contamination across conditions. Clinics will be randomized to one of the 16 conditions, with 12 ALHIV (aged 11–17 years) enrolled per clinic, yielding main effects and interaction effects for the 4 components on sustained viral suppression (primary outcome) [86]. Refer to Fig. 3 (SPIRIT flow chart).

### Description of four intervention components

#### Financial Literacy Training (FLT)

FLT is part of economic empowerment interventions that aim to infuse resources in poor families’ households, including a use of micro-savings, or offering financial literacy training. The focus is primarily on training, recognizing a problem several low-income families face: lack of business skills, personal empowerment, self-confidence, self-esteem and determination [87, 88]. The FLT workshops promote knowledge [87], because it is a critical element necessary in business success. For the Suubi intervention, FLT workshops will be implemented by community-level agencies in collaboration with local financial institutions via six workshops delivered over 6 weeks on weekends. FLT workshops will cover topics including: a) an introduction to the notion of asset-building; b) coverage of asset-building strategies in detail, e.g., saving; c) coverage of specific topics related to saving, e.g., the importance of saving and how to save, d) an introduction to banking services and e) coverage of basics of borrowing and debt management.

#### Incentivized matched Youth Savings Accounts (YSA) with income-generating activities (IGAs)

Matched youth savings accounts (YSA) go beyond incentivizing behavior—which is the mechanism of conditional cash transfers [89–94]. Matched YSAs promote savings habits and help establish and maintain partnerships between the participating family, local financial institutions and an intervention program. All YSAs are housed at a local bank and deposits made by the adolescent and family are matched by the intervention to encourage savings. YSAs introduce adolescents to formal financial institutions and incentivize saving by matching their deposits.

Participants will get a YSA held in their own name in a financial institution registered by the Central Bank (Bank of Uganda). Family members, relatives, or friends are allowed and encouraged to contribute to the YSA. The account is then matched with money from the program on a ratio of 1:2. The maximum matched amount is equivalent to 10 USD per month per family or 120 USD for a 12-month period. Families could also opt to use some of the money to meet school-related expenses (e.g. school lunch). A monthly bank account statement will be generated for each participant to note their accumulated savings. Statements are intended to act as “morale boosters.” During the intervention, each ALHIV and their primary caregiver as a co-signer will have access to the money in their account (excluding the matching funds). In emergency scenarios, for example, a family illness, participants may withdraw their own money—but not the matching funds [55, 62, 76, 95–98]. Matching funds are held in a separate account from the participants’ own savings. As part of YSA, ALHIV will also be trained on investing in income-generating activities (IGA) and are normally allowed to use up to 30% of their matched savings to invest in an IGA to benefit their family.

#### A manualized visual-based intervention for ART adherence and stigma reduction using multiple family group approach (Suubi Cartoon)

Suubi Cartoons are designed with principles from theories including: family systems theory, structural family theory and social learning theory with elements of psychoeducation and social group work [6, 99]. The cartoons adopted the therapeutic methods and theories to create a flexible approach that targets populations struggling with barriers to ART adherence [6, 53, 64, 66, 69, 76, 81, 99, 100]. The Suubi Cartoon curriculum describes the lead characters (Mabebeere and Kamperempe) learning about their HIV diagnosis and treatment needs, while coping with family loss, stigma, peer relationships, identity, and family functioning. The curriculum provides step-by-step guidance to deliver information and facilitate discussions and problem-solving within and between families. Adolescents together with her/his caregiving family, will attend sixteen 60 min sessions to be hosted within the community over 16 weeks. Each session will involve 6–10 families [2, 5, 6, 61, 63, 66, 101, 102]. Session topics include: 1) Knowledge about HIV/AIDS and STIs, 2) AIDS-related loss and bereavement; 3) HIV/AIDS stigma, discrimination and disclosure; 4) Youth identity, acceptance and coping with HIV; 5) HIV treatment and adherence; 6) Caregiver-child communication on sensitive topics (e.g., HIV, puberty); 7) Social support; 8) Identification of risk, alcohol, drugs, and peer pressure; 9) Self-esteem, negotiation, and refusal skills in risky situations; 10) Identifying/developing strategies to keep children safe in high-risk situations where sexual behavior and drug use are possible; 11) Puberty, ABC Model (Abstinence for those who can, being faithful and use of condoms), and protection from abuse.

#### Engagement with HIV treatment-experienced role models who share lived experiences of HIV.

ALHIV can benefit from engaging with role models (also known as treatment buddies) to identify specific future goals and aspirations through building their self-esteem, improving their HIV care engagement, reducing stigma and stress, encouraging hopefulness, building stronger communication skills with their caregivers and/or family members, enhancing safe sexual decision-making, and decreasing sexual risk-taking behavior. ALHIV will be placed into small groups with an average of 3–4 peers from the same health clinic, and each ALHIV stays in the same group for 9 sessions – with the same role model. This is intended to build trust and rapport not only between the role model and mentee, but also between all group members. The 9 sessions, will be conducted over a 6-month period, and includes activities, videos, scenarios, and role-playing to facilitate discussion and learning.

### Primary outcomes

We are measuring sustained viral suppression, which is a measure of viral load constancy.that is of great clinical and public health importance. Sustained viral suppression is defined as confirmed viral load laboratory tests of < 1000 copies per mL on all 12-, 24-, and 36-month follow-up assessments. Participants who have a viral load laboratory tests of > 1000 copies per mL at any follow-up assessment will be treated as not having sustained viral suppression. In line with another factorial experiment for viral suppression, missing viral load data and deaths will be treated as non-suppression and failure (details below). Participants who are virally suppressed on all 12-, 24-, and 36-month follow-up assessment are coded as 1 for sustained viral suppression. Participants who have missing data or are not virally suppressed on one or more 12-, 24-, or 36-month follow up assessment are coded as 0 for not sustained viral suppression. This handling of missing outcome data allows for calculating the differences in proportions between those who did and did not sustain viral suppression using an intent-to-treat approach [73, 103].

### Secondary and economic outcomes

We are using achievement of viral suppression as the outcome, defined as confirmed VL test < 1000 copies per mL at the 12-month follow-up assessment. This outcome was chosen because we aim to investigate the causal process and to meet the temporal sequencing assumption required for mediation and that any mediating effect, M, must occur prior to the evaluation of the outcome, Y, and after the delivery of each component, X. To meet the temporal sequencing assumption, we will collect data on all mediating variables during a second survey at 6-month assessment.

Cost-effectiveness will be determined using an activity-based ingredients approach to examine the costs of the component combinations to achieve a unit of effect across the primary outcome. The cost portion of the CEA will be measured during the 24 months that the intervention is delivered using the factorial design and the MOST framework.

### Eligibility

A total of 576 ALHIV (aged 11–17 years) will be recruited from 48 health clinics. Adolescents’ inclusion criteria are: 1) An adolescent living with HIV (confirmed by medical report and aware of status); 2) living within a family; 3) being 11–17 years of age (at enrollment); 4) Prescribed ART; and 5) enrolled in ART care at one of the 48 health clinics in the study districts. Health clinics would be eligible if they: 1) have existing procedures tailored to adolescent adherence (including adolescent-specific clinic days and peer counselling) and 2) accredited by the Uganda Ministry of Health as a provider of ART within the study districts. Exclusion criteria include an adolescent’s inability to understand study procedures and participant rights as assessed during informed consent/assent process with the adolescent or parent. If the adolescent or adult caregiver presents with emergency needs (e.g., hospitalization), needed care will be secured, rather than study participation.

### Screening and enrollment at sites

Using the same recruitment procedures used in preliminary studies (Suubi + Adherence R01HD074949), participants will be screened and recruited from 48 health clinics in six districts in Southern Uganda. Participants will be identified and recruited from the healthcare clinics associated with ICHAD, RTY-Uganda, and Masaka Diocese. Patients are seen at least annually and each patient on ART must have prescriptions filled monthly at the hospital/health clinic pharmacy [104]. Although appointment days (not times) are provided, most patients usually arrive early in the morning on days that are convenient for them and wait for several hours before they are seen. This provides an opportunity for recruitment through medical staff. The medical staff will create a list of all eligible families using medical records [104]. Each medical chart contains data on each patient’s HIV status and age, and family data. A clinic staff member will review the daily schedule of patients, and indicate to providers which patients are the eligible [104]. Providers will then present the project to caregivers of eligible adolescents during appointments. If caregivers are interested, verbal consent to be contacted by research staff (who will be on-site during clinics) will be requested [104]. After speaking with the research Project Coordinator about the study, interested caregivers will provide written consent for adolescent participation. Adolescents will be asked for written assent. If caregivers are not at the medical appointment, a community health worker from the clinic will outreach to parents and caregivers in the community. Given the cultural context within which the proposed study will be undertaken, if multiple adolescents in a family are eligible, all will be recruited if they meet the inclusion criteria [104]. This is intended to avoid any kind of envy or resentfulness that may occur when other adolescents in the family who meet the inclusion criteria were to be excluded [104].

### Contamination across conditions

Randomization to any one of the 16 conditions occurs at the level of the health clinic such that all adolescents from the same clinic receive the same intervention component to avoid contamination. The health clinics located in six districts in Southern Uganda are widely spread and far apart, in distance, from each other. Further, we will train all staff to encourage all families and participants to not discuss their intervention assignment to others if possible.

### Survey and clinical data collection

Survey assessments will occur at baseline, 6-, 12-, 24-, and 36-months, and all assessments will take place in ICHAD’s private research field offices in Masaka; at satellite sites (MildMay, RTY- Uganda); or at the participants’ homes (if they request it and there is sufficient privacy to ensure confidentiality, or at clinics (in a private room) with each lasting about 60 min [105]. Flexibility in interview location has been critical to Suubi + Adherence and other ICHAD studies success to date, with approximately 40% conducted in research offices in Masaka, and 50% in satellite sites [105]. Assessments are administered orally however for questions measuring sensitive behaviors, computer assisted self-interviews will be administered [106]. Non-sensitive questions will be interviewer-administered using Qualtrics [106]. Viral load assessments occur at the same time points but not at the 6-month follow-up assessment. Surveys will be conducted in English or Luganda depending on participants’ English proficiency [106]. All interviewers will be fluent in both languages. In Table 1, we provide a list of standardized instruments that will be included in the main statistical analyses. All measures used have been or will be pre-tested and made appropriate for the local Ugandan context [106].

**Table 1:** Survey, clinical, and costs data

**Variables Moderators**	**Measurement**	**Time Point (Months)**
1. Demographics 2. Age 3. Sex (assigned at birth) 4. Family: Orphan status, socioeconomic status, family composition/structure, caregiver educational level 5. Geographic location and exposure to outside HIV-related programs	Self-reported Questionnaire	Baseline, 6, 12, 24, 36
**Mediators and Moderators**		
1. Financial literacy; 2. Financial management skills; 3. Engagement with financial institutions	Self-reported questionnaire Data from Bank Statements Self-reported questionnaire	6 months
1. Knowledge about ART 2. Internalized stigma 3. Sexual negotiation and communication skills 4. Mental health and HIV identity	Self-Report Questionnaire and Pill counts Social impact scale [107] Sexual Communication Scale [108] Condom negotiation scale [109] Beck Hopelessness Scale [110] and Center for Epidemiological Studies-Depression Scale (CESD) [111] & Negative Self-image sub scale of HIV Stigma Scale, [112] HIV-Positive Identity Questionnaire [113]	6 months
1. Problem solving of HIV care logistics 2. Self-esteem and social support 3. Engagement and retention in HIV care	Self-reported questionnaire Rosenberg Self-Esteem Scale [114] and Social Support Scale [115] HIV Index of Engagement [116, 117] and clinic records	6 months
**Primary Outcomes**		
Viral load		Baseline, 12, 24, 36
**Costs Data**		
Costs for staff time, supplies, overhead for IDAs	Project records, administrative review	(cost data will be collected during the 24 months that the intervention is delivered)

### Fidelity monitoring for interventions and quality assurance of study data

To ensure safety and protection of participants, we will conduct extensive research assistant (RA) training prior to study implementation. RAs employed in Uganda will have experience conducting interviews and data collection with vulnerable populations in Uganda. All will complete Human Subjects training as well as training on good clinical practice. Additionally, each of the RAs (to be hired) will be fluent in both English and Luganda, the local language that will be used in the study. RAs will have the ability to follow written and oral directions, be amenable to being evaluated, and have interpersonal flexibility. Research Assistant training (quantitative) will include (1) ethical issues in research; (2) establishing and maintaining rapport with participants; (3) obtaining informed consent; (4) addressing participants’ concerns with confidentiality and handling sensitive situations; (5) monitoring interviews; (6) obtaining accurate tracking information; (7) managing distress and conducting crisis and enhanced referrals; (8) detecting, handling, and reporting adverse events; and (9) working appropriately within host agencies/study sites. In addition, the performance of each RA (e.g., recruitment/refusal rates, data recording/entry errors) will be monitored; should an interviewer consistently perform at a level below other RAs for a 30-day period, he or she will be required to repeat the RA training.

Facilitator training will include (1) ethical issues in research; (2) establishing and maintaining rapport with participants; (3) addressing participants’ concerns with confidentiality and handling sensitive situations; (4) conducting their assigned intervention; (5) managing distress and conducting crisis and enhanced referrals; (6) detecting, handling, and reporting adverse events; and (7) working appropriately within host agencies/study sites. Intervention training will require pre-training preparation, asking RAs to review the protocol describing the goals, purpose, and design of the study, the theoretical framework, intervention manuals, materials, and a few key selected readings on theoretical concepts underpinning the intervention and to conduct mock sessions with trainers and peers. Facilitators will receive feedback from each other and the trainers and fashion common responses to issues that may arise during sessions. Facilitator training will stress the importance of implementation fidelity to study success. MPIs will certify facilitators prior to their study intervention facilitation. Certification requires satisfactory demonstration of key intervention activities and components.

For data management, the Management Information System for Individual Development Accounts Quality Control (MIS IDA QC) software [71] will be used to check for data-entry errors and prevent missing values. Frequency tables for all variables and measures of central tendency and variability for continuous variables will characterize the sample overall. Missing data will be addressed with direct maximum likelihood (ML) and multiple imputation (MI) [118] under the conditionally missing-at-random (MAR) mechanism [119]. Auxiliary variables will be included to help meet the MAR assumption [120, 121] and sensitivity analyses will be conducted with pattern-based MI [122] to assess the robustness of the MAR assumption [120, 123–127].

Secondary outcome, ART Adherence will be measured through self-report by three items: 1) frequency of pill taking, 2) ability to take pills, and 3) missed doses in the past 4-weeks and 6-months [128]. This will be augmented with a comprehensive unannounced pill count method that includes home visits, and ascertain each participant’s regimen before actual counting which would be done in the presence of the participant. Participants will be visited at an undisclosed time and day to count their pills once every 3 months throughout the study period.

### Follow-up plan and tracking

The project will take place in a highly stable region of Uganda where mobility is rare. We will collect telephone numbers, names, addresses, and contact information for three family/friends who know of each participant’s location. We will also be in contact with all participants regularly to determine enrollment and attendance. Moreover, we will have monthly contact with participants across the study conditions through the pill counting (see details above). These procedures will enable our team to continually engage all participants, and minimize loss to follow-up. These strategies were used in our earlier NIH funded studies [2, 53, 55, 56, 63, 65–67, 129–131] including the 6-year *Suubi* + *Adherence* study [104] which yielded a 93.4% retention rate over a 6-year period. Given these numbers and our team’s experience in the study region, we conservatively expect attrition by end of follow-up to be no more than 20%.

### Data sources

Data for the proposed study will be obtained through an eligibility screening checklist, and computer assisted, interviewer-administered structured questionnaires. Self-reported data via interviews will be collected at baseline and at all follow-up assessments from all participants. We will use self-report questionnaires with read-aloud procedures by professional trained staff to address issues of literacy. Multiple research assistants (RAs) will be hired to conduct assessments. To encourage truthful responding, we will remind participants that responses are confidential and explain to them the security system that makes it impossible to link a name to data. Each participant will complete a total of five interviews (one at baseline, then four follow-up interviews). One at 6 months (after delivery of intervention) then three 12 months apart (from intervention initiation).

The battery takes into account: 1) sensitivity to participant’s literacy (RAs read questions aloud and help fill out measures); 2) need for trust and rapport, and 3) use of local phrases and terms. In our previous work, participants completed these interviews without incident, often reporting positive experiences in being interviewed. Moreover, interview breaks and snacks will be provided and sensitive interviewers who are familiar with interview questions will facilitate the process.

### Harms

There are no major risks involved, however, a participant may feel embarrassed or uncomfortable during the consenting and interview process when answering sensitive and personal questions, taking blood for viral load testing. The process of blood draw may cause some discomfort, bleeding, or bruising where the needle enters the body, and in very rare cases, fainting or infection. First and foremost, interviewers will make it explicitly known to the participants that they may refuse to answer a question or decline to undergo a procedure, at any time. This will also be explained in writing on the consent forms. If a participant tells the interviewer that he/she is uncomfortable with a particular topic, that he/she prefers not to discuss a particular topic or feels he/she cannot participate in the biomarker process, the interviewer will move on to the next question/part of the interview.

### Data analysis plans

Effect coding is used to produce uncorrelated main effect estimates, unbiased standard errors, and clearer interpretations of interactions between components in factorial designs. The single covariate of randomization stratum with four levels reflecting the combination of two binary variables: 1) ALHIV population size (medium size vs. large) and 2) geographical location (rural vs. urban), to ensure balance on those variables. Our preliminary analysis will focus on assessing balance in assignment to conditions. The stratified restricted cluster randomization is expected to produce balance across the 16 conditions [132]. We expect to have equal cell sizes to estimate the main effects and interactions and equal percentages of ALHIV population sizes and geographical locations in our main comparisons. For thoroughness, we will check for imbalances across the four component comparisons (e.g., percent of rural vs. urban participants in the financial literacy versus no peer financial literacy comparison), rather than across 16 conditions because recall that a 2^4^ factorial design is not a 16-arm comparison. In the unlikely event that an imbalance does occur by ALHIV population size and/or geographic location, we will use propensity score matching (i.e., a conditional probability of having one component delivered versus another) with baseline covariates collected in our survey to estimate the primary outcome under the counterfactual assumption of balanced groups [133–135]. The proposed analyses and syntax code will be documented and made available on request to enable review, transparency, and results reproducibility.

The goal is estimating main effects for sustained viral suppression. Factorial designs with continuous, normally-distributed outcomes measured cross-sectionally are often evaluated using an Analysis of Variance (ANOVA), which is a special case of a general linear model (GLM). For.analyses with clustering (within-subject correlations) and non-normal outcomes (e.g., sustained viral suppression), generalized estimating equations (GEE) can be used instead of ANOVA [136–138]. Recall that our primary outcome is a single computed variable of sustained viral suppression—all 1 s on the Y variable at 12-, 24-, and 36-month follow-up assessments. Thus, we are using GEE, not based on repeated measures across time, but because of clustering that will occur through the cluster randomized trial (CRT) design approach we are using. Participants who visit the same clinic may not be statistically independent [137]. Thus, GEE can properly account for the correlation of participants within the same clinic by using robust standard errors to correct inferences, even if the chosen correlation structure remains slightly misspecified. Moreover, within-clinic correlations of outcomes are considered nuisance parameters, not quantities of interest to be modeled explicitly [136]. In GEE, we will a priori specify a working correlation structure—specifically the exchangeable correlation structure (compound symmetry assumption)—given the assumption of a balanced factorial design and a single level of clustering by clinic [139]. Alpha (α) will be set at 0.05 for estimating main effects, which are orthogonal (i.e., independent) of each other. As detailed by Kahan and Morris [140], stratification during the randomization process must be accounted for in the primary analysis as it may lead to correlations among the participants in each cell. Uncorrected, this would bias standard errors estimates, producing wider confidence intervals, a lower Type I error rate, and affect statistical power. Thus, all analyses will be adjusted by randomization stratum to produce unbiased estimates.

Factorial designs are innovative in their ability to estimate how two or more components interact, which is not possible with a multi-component packaged intervention. Following the MOST framework, α will be set to 0.10 for all interaction effects, which is justified by the decision-priority perspective [141] guiding this study. To enhance rigor, we will test if interactions among components yield additive and/or synergistic interactions. Additional exploratory analyses will add sex as a covariate and extend each of the previously-described analyses to include interactions of each effect with sex. We propose the following hypotheses for the mediation analyses (also depicted in conceptual model, Fig. 2).

### Data analysis plan for secondary and economic outcomes

For rigor, mediation will be tested as the product of the a-path coefficient (*a* = effect of X on M) and b-path coefficient (*b* = effect of M on Y). Additionally, the diagnosis of weak performing components and high-performing components will be determined by evaluating separate paths (a-path = X to M, b-path = M to Y). We will estimate the a-path, b-path, and indirect coefficients and their 95% confidence intervals via causal mediation approaches in M*plus,* which allows binary mediators and/or outcomes [142, 143] and can compute cluster-adjusted hypothesis tests for mediators, which is important given our study’s cluster-randomized design [144]. Reflecting the decision-priority framework and to minimize Type II error, α will be set at 0.05 for hypothesis tests. Mediators will be measured at the 6-month follow-up period to ensure the required ordering of variables.

Secondary exploratory analyses include identifying moderators of the intervention component-viral suppression relationship. Based on the *principle continual optimization*, moderation analyses allow understanding of *when* and *among what person characteristics* the component creates change. The optimized intervention can then be refined based on effect sizes that change based on a moderator of the component-outcome relationship. Potential clinic and participant moderators are shown in our conceptual model depicted in Fig. 2 and include variables such as sex, age, geographic location, ALHIV population size, HIV acquisition mode, and exposure to HIV/STI programs external to the study. Significant interactions will be further probed using simple main effects for categorical moderators and, for continuous moderators, changes in component-outcome effects at percentiles of moderators plus the Johnson-Neyman method [145]. Finally, additional exploratory analyses will assess moderated mediation by employing causal mediation methods to decompose the total component-outcome effect to evaluate whether the effect of the intervention component on the outcome is due to mediation alone, moderation alone, mediation and moderation together, or neither. Reflecting our decision-priority framework, tests of moderated mediation will be evaluated at α = 0.10.

### Economic evaluation

Following standard practice, we will measure costs for each of the 16 combinations on a per-person basis using an activity-based approach. Costs of the interventions include all program costs, such as YSA savings match, costs for the FLT workshops, Suubi Cartoon, engagement with role models, training, volunteers and donated materials, and running the programs. Research costs are excluded. Costs will be divided by effects to determine efficiency in CEA. To compare the value of two mutually exclusive approaches, a typical RCT reports the cost per added gain as the incremental cost-effectiveness ratio (ICER = (C1-C0)/(E1-E0)), where C=cost, E=effect, 1=intervention, and 0=baseline. The ICER is often compared to other such estimates in the literature and to a decision-maker’s threshold of acceptability to guide policy. For MOST and the factorial design, CEA decisions and optimization are more complex [146]. First, we will test whether any of the 4 components have interacting costs or effects—i.e., economies or effects of scale (If there are no significant interactions, decisions to adopt each of the combinations can be made independently). Next, interventions will be ordered by increasing cost, and we eliminate any “dominated” combinations which are more costly but less effective than a cheaper strategy. Finally, we will compute ICERs for comparison to the literature (such as our past studies [77, 78]) and for assessment as policy options, such as comparing to pre-exposure prophylaxis. Our baseline CEA will focus on ICERs with no budgetary threshold. Next, we will use the MOST framework to assess ICERs while placing constraints on affordability and scalability to select the best possible combination for various levels of a decision-maker’s preferences for affordability and scalability alongside efficiency. Confidence intervals will be generated using bootstrapping and Monte Carlo simulations.

### Power analysis

Power analyses for our proposed primary analysis was generated using the multilevel logistic regression module for proportions in a 2-level hierarchical design in NCSS PASS 21 [147]. We set the base rate of sustained viral suppression to 50% based on MPI Ssewamala’s Suubi-Adherence study. We assumed *N* = 576 participants recruited from 48 clinics and set α = 0.05, power = 0.80, and the intracluster correlation (ICC) due to clustering of participants within clinics at 0.024 based on data from MPI Ssewamala’s Suubi-Adherence study. We conservatively assumed 20% attrition (*N* = 480). The minimum detectable odds ratio is 1.77, which corresponds to a minimum raw proportion difference of 14% and a standardized effect size of 0.28, which is between a small to medium effect size. For Specific Aim 2, the only difference in power calculations was we set the base rate of sustained viral suppression to 75% and the ICC to 0.038 based on 12-month achieved VL suppression data from MPI Ssewamala’s Suubi-Adherence study. Under these conditions, the minimum detectable indirect effect odds ratio is 1.80, which is between a small and medium effect size [148].

### Determining the Optimized Intervention

The operational definition of an optimized intervention is one that produces the most cost-effective outcome while meeting the optimization objective, the most cost-effective outcome under our pre-defined real-world constraints (i.e., efficiency, affordability, and scalability). Within the MOST framework, there are two key perspectives to how determine optimization. First, in a *conclusion-priority perspective*, optimization is based solely on the statistical significance at *p* < 0.05 of each component (rejection of the null hypothesis) [141]. However, if a component or an interaction effect failed to reject the null hypothesis, it is not known whether that component or that interaction genuinely had an effect on viral suppression or if a Type II error (incorrect rejection) was made. This perspective (i.e., *p* must be less than 0.05 alone) may erroneously suggest that one or more components should not be included in the optimized intervention. In a *decision-priority perspective* [141], the components and interactions that evidence a statistically significant effect may or may not be included in the optimized intervention as their inclusion depends on the optimization objective – most cost-effective outcome under our pre-defined real-world constraints (i.e., efficiency, affordability, and scalability). For example, our primary analysis will identify the combination of components that produce the greatest odds for sustained viral suppression at *p* < 0.05. However, regarding optimization, if the difference in the odds between having 3 and 4 components is 5%, we could evaluate whether that difference is worth the burden, time, or cost to implement the fourth component. Or, three components could synergistically interact and produce an effect equivalent to having all four components delivered. In a decision-priority perspective, optimization does not have to be based on statistical significance alone but on identifying the costs, effect sizes, and feedback from staff and investigators on the best number of components for sustained viral suppression among ALHIV.

## Discussion

The Suubi + Adherence4Youth study is following the shift in the paradigm of research to use new thinking to build/un-pack highly efficacious interventions that lead to new scientific knowledge in terms of understanding what drives an intervention’s success and how to iterate on them in ways that are more efficient, affordable and scalable [73]. The concept and methods proposed to critically reflect and test our prior Suubi intervention advances intervention science during a critical period of time for a vulnerable group – as ALHIV transition through young adulthood. Most HIV prevention, care and support efforts in SSA communities are “transported” from outside the region, mainly from the global north [1–7], and offer limited information in understanding why or how they are affected by the low-resource context. Yet, this knowledge is imperative, and it is important to note that the Suubi intervention strategies are developed and tested within the global south’s existing institutions and infrastructure. Moreover, Suubi + Adherence4Youth has additional important advantages: it is theoretically driven; comprehensive, and carries a range of contextual and modifiable components that make it ideal for scale up. We are addressing structural economic factors that constitutes a major gap in HIV intervention science, especially in the context of resource-constrained settings. Most existing interventions were developed and implemented in high-income developed societies with sufficient human and financial resources available to support well-functioning social welfare systems [104]. As a result, in poorer countries where resources are limited, dissemination of such interventions are restricted. Among 14 intervention studies reviewed, findings showed that participants’ adherence improved slightly in nine studies, while in three intervention effects were sustained over time [149, 150]. However, the majority of interventions utilize cognitive behavior models [149, 150] and thus there are difficulties in sustaining the effects of psychosocial interventions over time.

Thus, while short-term psychosocial interventions have become an accepted strategy to manage the staggering numbers of HIV patients—registering small to moderate effect sizes at best [149, 150]– a failure to explicitly address economic hardships will limit the degree to which we can meaningfully improve HIV care outcomes. The Suubi + Adherence4Youth study is primarily interested in viral suppression via economic and psychosocial support systems. In the context of resource-poor countries, interventions that improve families’ economic capabilities are likely to be particularly consequential. This study will employ a highly efficient MOST framework that will allow us to understand the role each component in the Suubi intervention plays in producing the outcome across 48 health clinics in Uganda for a large sample of ALHIV. This is innovative because the approach may yield an optimized intervention that can be progressively refined for other resource constrained countries in SSA—one of the world’s poorest regions with the highest HIV prevalence. Taken as a whole, this innovative approach can pave the way for other researchers to build directly from our conceptual model and evidence base in a way that is transparent and forward thinking. The proposed study offers us an opportunity to cost each of the four components and every combination of Suubi components to achieve a unit of effect across the primary outcome. This may offer insights in regards to potential for scale-up in real-world constraints.

## Supplementary Information

**Additional file 1. **SPIRIT Checklist

## Data Availability

Not applicable.

## Abbreviations

AIDS: Acquired Immunodeficiency Syndrome
ALHIV: Adolescents living with HIV
ANOVA: Analysis of Variance
ART: Antiretroviral Therapy
CEA: Cost-Effectiveness Analysis
CRT: Cluster Randomized Trial
DSMB: Data and Safety Monitoring Board
FLT: Financial Literacy Training
GEE: Generalized Estimating Equations
GLM: General Linear Model
HIV: Human Immunodeficiency Virus
ICC: Intracluster correlation
ICER: Incremental Cost-Effectiveness Ratio
ICHAD: International Center for Child Health and Development
IGA: Income-Generating Activities
IRB: Institutional Review Board
MAR: Missing-At-Random
MI: Multiple Imputation
MIS IDA QC: Management Information System for Individual Development Accounts Quality Control
ML: Maximum Likelihood
MOST: Multi-phase Optimization Strategy
MPI: Multiple Principal Investigator
NIH: National Institutes of Health
RA: Research Assistant
RCT: Randomized Controlled Trial
RHSP: Rakai Health Sciences Program
RTY-Uganda: Reach the Youth Uganda
SSA: Sub-Saharan Africa
UNCST: Uganda National Council of Science and Technology
USD: United States Dollar
UVRI: Uganda Virus Research Institute
WUSTL: Washington University in St. Louis
YSA: Youth Savings Accounts
